# Finding the superior allele of *japonica-*type for increasing stem lodging resistance in *indica* rice varieties using chromosome segment substitution lines

**DOI:** 10.1186/s12284-018-0216-3

**Published:** 2018-04-18

**Authors:** Indria Wahyu Mulsanti, Toshio Yamamoto, Tadamasa Ueda, Ahmad Fahim Samadi, Eri Kamahora, Indrastuti Apri Rumanti, Vo Cong Thanh, Shunsuke Adachi, Sakae Suzuki, Motoki Kanekatsu, Tadashi Hirasawa, Taiichiro Ookawa

**Affiliations:** 1 United Graduate School of Agricultural Science, Tokyo University of Agriculture and Technology, 3-5-8 Saiwai-cho, Fuchu, Tokyo, 183-8509 Japan; 2 Institute of Crop Science, NARO, 2-1-2 Kannondai, Tsukuba, 305-8666 Japan; 3 Indonesian Agency for Agricultural Research and Development-Indonesian Center for Rice Research, Subang, 41256 Indonesia; 4 College of Agriculture and Applied Biology, Can Tho University, Campus II, 3/2 street, Ninh Kieu, Can Tho, Vietnam

**Keywords:** Bending-type lodging, Breaking-type lodging, Cell wall material density, QTL, Rice

## Abstract

**Background:**

In cereal crops, stem lodging can be classified into two types: stem-breaking type and stem-bending type. To improve stem-lodging resistance, the strong culm traits of superior lodging-resistant varieties must be characterized. The identification of quantitative trait loci (QTLs) and the corresponding genes associated with the parameters for bending moment at breaking (M) and flexural rigidity (FR) is expected to enable the efficient development of lodging-resistant varieties. A set of Chromosome Segment Substitution Lines (CSSLs) derived from the cross between Takanari and Koshihikari were used in this study to identify QTLs associated with lodging resistance.

**Results:**

The *indica* variety Takanari possesses large M due to its large section modulus (SM) despite its small bending stress (BS), whereas Takanari also has large FR due to its large secondary moment of inertia (SMI) and Young’s modulus (YM). The QTLs for BS were assigned to chromosomes 3, 5, 6, 8, 9, 10, 11, and 12. Koshihikari alleles increased BS in these QTLs. The YM was increased by substitution of the Koshihikari chromosomal segments on chromosomes 2, 10, and 11. Other QTLs mapped to chromosomes 7 and 12, such that the Koshihikari alleles contributed to the decrease of YM. QTLs for cellulose density were assigned to chromosomes 1, 3, and 5, which were replaced by substitutions of Koshihikari segments. The QTLs for hemicellulose, cellulose, and holocellulose densities identified on chromosome 5 overlapped with those for BS, indicating the positive effect of the Koshihikari segment on increasing BS.

**Conclusions:**

These results suggested that the QTLs for the densities of cell wall materials in *japonica* varieties contributed to increased BS and might be utilized for improving lodging resistance in *indica* varieties of rice.

**Electronic supplementary material:**

The online version of this article (10.1186/s12284-018-0216-3) contains supplementary material, which is available to authorized users.

## Background

Lodging has been an important constraint on rice production in monsoon Asia. When lodging occurs after a typhoon hits, the canopy structure is destroyed, and the capacities for photosynthesis and dry matter production are sharply reduced. In severe cases, lodging can result in breaking of the stem or pulling out the roots, blocking the transport of water, minerals, and photoassimilates and leading to declines in yield and quality.

The introduction of dwarfism in rice, which led to the green revolution in the 1960s, effectively increased lodging resistance and increased the harvest index of rice (Khush 1999). Reduction of plant height became a goal for improving lodging resistance in plant breeding. However, the improvement of lodging resistance in rice by alterations to the *semi-dwarf1* gene (*sd1*) alone has reached a limitation. This plateau reflects the fact that decreases in total biomass production potentially disrupt the balance between the source and sink capacities, as in the case of dwarf rice plants with diminished grain yields. Another problem with semi-dwarfism is that *sd1* has negative pleiotropic effects on culm morphology (Ookawa et al. 2010b). For example, mutation of *sd1*, which encodes a gibberellin (GA) biosynthetic factor, also reduces culm strength by decreasing culm diameter and thickness (Okuno et al. 2014). Because of these pleiotropies, using semi-dwarf genes to increase lodging resistance is difficult. In gramineous crops, the nature of lodging is closely related to the anatomical features (Kong et al. 2013; Matsushita et al. 2014; Wu et al. 2011) and cell wall components (Yang et al. 2009; Ookawa and Ishihara 1993) of the culm. Some studies report that culm strength is significantly correlated with culm cellulose and hemicellulose contents (Kokubo et al. 1991; Li et al. 2003). Other studies show that lignin also contributes to culm strength (Li et al. 2003; Jones et al. 2001).

According to Sterling et al. (2003), lodging in cereal crops can be classified into stem lodging and root lodging. Stem lodging can be subclassified into stem-breaking type and stem-bending type. Stem-breaking lodging results from excessive bending pressure at the basal internode and is determined primarily by the morphology and quality of culm (Ma et al. 2003; Zuber et al. 1999; Ookawa and Ishihara 1992). Stem-bending type lodging occurs in the entire internode when the stem cannot withstand the bending pressure (Islam et al. 2007).

In breaking-type lodging, the bending moment at breaking (M) of the basal culm is a critical trait for resistance to lodging (Wu et al. 2011; Ookawa et al. 2010a). The M is divided into two components, section modulus (SM) and bending stress (BS). The SM is determined by culm diameter and culm wall thickness (Zang et al. 2016; Hirano et al. 2014), whereas the BS is related to parameters such as the morphology of cortical fibre tissue (Ookawa et al. 2014, 2016) and the densities of cell wall materials (Ookawa et al. 2014; Yang et al. 2009; Ookawa et al. 1993).

Bending-type lodging occurs at the beginning of lodging and triggers breaking-type lodging. Bending-type lodging is observed in the entire internode of rice during strong wind and rain (Ishimaru et al. 2008; Sterling et al. 2003). The process is associated with heavy panicle, weak culm, and higher plant stature (Kong et al. 2013; Chuanren et al. 2004). The flexural rigidity (FR) of the culm affects lodging resistance (San-Oh et al. 2001). FR is the product of Young’s modulus (YM) and the moment of inertia of area about the neutral axis (Silk et al. 1982). A previous study showed that Nipponbare, a *japonica* variety, had a small FR due to a small secondary moment of inertia (SMI) and that a large FR value reflected a large YM (San-Oh et al. 2001). Based on that study, a large SMI and YM are important for increasing the FR of the culm.

An improvement of lodging resistance in rice could be achieved by increasing culm stiffness such as BS, which is attributed primarily to high cellulose and lignin content (Ma et al. 2000; Yang et al. 2009; Ookawa et al. 2014). In previous studies, the Koshihikari possessed a small SM (indicative of culm thickness), whereas also displaying a large BS (indicative of culm stiffness) (Ookawa et al. 2010a). Furthermore, the Koshihikari has thick cortical fibre tissue (Ookawa et al. 2016). These traits are responsible for the high BS in Koshihikari (Ookawa et al. 1993). In contrast, Takanari, an *indica*-type variety, showed a large SM due to a large outer diameter and a small BS due to a thin cortical fibre tissue (Ookawa et al. 2016). However, whether the cell wall components contribute to the difference in BS has been clarified. To improve the stem lodging resistance in *indica* rice varieties, identifying the quantitative trait loci (QTLs) for these traits is necessary because resistance results from quantitative traits controlled by multiple genes.

Chromosome Segment Substitution Lines (CSSLs) are powerful tools for identifying the QTLs for agronomic traits (Ali et al. 2010). CSSLs carry specific donor chromosome segments in the genetic background of recurrent varieties and have been used to detect QTLs with large and small effects that often are obscured by QTLs with large effects. The utilization of CSSLs permits the detection of QTLs distributed across the genome, despite requiring fewer plants than those required for other techniques such as F_2_ or RILs (Kubo et al. 2002; Ebitani et al. 2005; Ando et al. 2008; Abe et al. 2013). To identify and facilitate genetic analysis of complex traits in rice, a series of CSSLs have been developed (Furuta et al. 2014; Ando et al. 2008; Takai et al. 2007; Xi et al. 2006). An example of the application of this technique to lodging resistance is provided by the study of Ookawa et al. (2010b), which used *indica*-type Habataki CSSLs in a *japonica*-type Koshihikari genetic background to demonstrate that *ABERRANT PANICLE ORGANIZATION1* (*APO1*) (previously reported to control panicle structure) increases culm strength by increasing culm thickness.

Reciprocal CSSLs between Koshihikari and the *indica*-type high-yielding variety Takanari have been developed to precisely identify QTLs for important agronomic traits (Takai et al. 2014). Recently, a study by our group identified QTLs associated with the thickness of cortical fibre tissues, showing that these QTLs are located on chromosomes 2, 9, and 11 (Ookawa et al. 2016). However, the QTLs for cell wall components associated with culm stiffness such as BS have not been reported. In the previous study (Ookawa et al. 2016), the Takanari line has a large SM that contributes to M, although the Takanari had a small BS. M reflects breaking-type lodging resistance. The substitution of the corresponding segment from Koshihikari into the Takanari genetic background is expected to contribute to an increase in BS and M. The introduction of a superior allele from a *japonica* variety with weak culm might be utilized for improving lodging resistance in an *indica* variety.

The purpose of the present study was to identify important traits of bending- and breaking-type lodging resistance; to estimate related QTLs using CSSLs derived from a cross between *indica*-variety Takanari and *japonica*-variety Koshihikari; and to find the hidden superior allele of the *japonica* variety that can increase the lodging resistance of the *indica* variety.

## Methods

### Plant material and cultivation

Thirty-seven Koshihikari CSSLs in the Takanari genetic background (T-CSSLs) that were derived from a cross between Takanari (*Oryza sativa* L. spp. *indica*) and Koshihikari (*Oryza sativa* L. spp. *japonica*) were used for the estimation of QTLs (Additional file 1: Figure S1). The parents, which served as controls, were planted under the same conditions as the CSSLs in 2015. To confirm the region of QTLs responsible for physical parameters and cell wall materials on chromosome 5, reciprocal CSSLs were analysed in 2016 (Additional file 2: Figure S2).

Rice seeds were sown in nursery boxes. Seedlings were transplanted, at a density of one plant per hill, to a paddy field at the university farm in Tokyo on alluvial soil of the Tama River. The planting density was 22.2 hills m^− 2^, with a spacing of 15 cm × 30 cm. As a basal dressing, compound fertilizer was applied at a rate of 5.0 kg 10 a^− 1^ for N and 6.0 kg 10 a^− 1^ for P_2_O_5_ and K_2_O.

### Measurement of culm strength

Morphological characteristics and breaking strength were measured at the basal internode. Six culms from each plot were measured at 14 days after heading. M and YM were measured at a distance of 4 cm between supporting points by the method of Ookawa and Ishihara (1997) using a Tensilon RTG-1210 universal testing machine (A&D, Tokyo, Japan).

The physical parameters of culm strength were used for precise phenotyping of breaking-type and bending-type lodging resistance. Physical parameters for breaking-type lodging resistance were calculated using the following formulas:

$$ \mathrm{M}=\mathrm{SM}\times \mathrm{BS} $$

$$ \mathrm{SM}=\left(\frac{\pi }{32}\right)\times \left({a}_1^3{b}_1-{a}_2^3{b}_2\right)/{a}_1 $$

where **a1** is the outer diameter of the minor axis in an oval cross-section, **b1** is the outer diameter of the major axis in an oval cross-section, **a2** is the inner diameter of the minor axis in an oval cross section, and **b2** is the inner diameter of the major axis in an oval cross section.

In bending-type lodging resistance**,** FR describes the resistance to bending. FR is the product of Young’s modulus (YM) and the secondary moment of inertia (SMI). SMI and FR were calculated using the following formulas:

$$ \mathrm{SMI}=\left(\frac{\pi }{64}\right)\times \left({a}_1^3{b}_1-{a}_2^3{b}_2\right) $$

$$ \mathrm{FR}=\mathrm{YM}\ \mathrm{x}\ \mathrm{SMI} $$

### Determination of cell wall materials

Dried, ground culm samples were treated at 80 °C for 20 min with 80% ethanol; the treatment was then repeated with 50% ethanol. The pellet obtained after the second ethanol extraction was further treated with heat-stable *Bacillus licheniformis* α-amylase (12 mg powder / ml in 200 mM Pi buffer; 600 U in a reaction volume of 100 μl) to remove starch. After incubation at 85 °C for 30 min, the samples were centrifuged, the supernatants were discarded, and the pellets were washed with deionized water. Pellets were dried overnight at 80 °C and then cooled in a desiccator with repeated weighing until a constant weight was obtained (i.e., to perfection). For the determinations of holocellulose and hemicellulose contents, lignin was degraded by resuspending the starch-free residue in 1.6 ml of NaClO_2_ (400 mg NaClO_2_ / 60 ml of deionized water) and 0.2 ml of 99.7% acetic acid (CH_3_COOH), followed by incubation of the suspension at 80 °C for 60 min. The samples were centrifuged, the supernatants were discarded, and the treatment was repeated one more time. The resulting pellets were washed twice with deionized water and once with acetone and then dried to perfection. To permit determination of the cellulose and hemicellulose contents, the sample was subjected to acid detergent extraction of bulk hemicelluloses. Specifically, 600 μl of acid detergent solution was added to the dried pellet, and the mixture was incubated at 95 °C for 1 h to hydrolyse the hemicelluloses and separate them from the remaining cell wall components (cellulose). The resulting pellet was washed with deionized water and acetone and then dried to perfection.

### QTL estimation by CSSLs

When the average value of a trait was significantly different between a CSSL and its recurrent parent, the QTL’s existence was estimated according to Dunnett’s multiple comparison test. QTLs were assigned to the substitution chromosomal segment based on the identity of CSSLs deduced in Ando et al. (2008). In the present study, QTLs for the strong culm trait and cell wall materials content associated with lodging resistance were detected using T-CSSLs. Reciprocal T-CSSLs and K-CSSLs (CSSLs with Koshihikari genetic background) on chromosome 5 were used to detect overlapping QTL regions.

### Statistical and QTL analyses

Statistical comparison of multiple sets of data was conducted using Dunnett’s multiple comparison tests with R software version 3.4, and a *t*-test was used to test the differences between parents. Means between parent and CSSLs on chromosome 5 were analysed using a *t*-test when ANOVA showed significance at the level of 0.05 probability. ANOVA tests were conducted using Jmp software ver.12.0.1. ANOVAs for the influence of lines on breaking-type lodging and cell wall composition densities of chromosome 5 are presented in Additional file 3: Table S1.

## Results

### Breaking- and bending-type lodging resistance in Takanari and Koshihikari lines

Large differences were observed in the parameters of both breaking- and bending-type lodging resistance in 2015 and 2016 (Table 1). The M of Takanari was larger than that of Koshihikari because of the larger SM. The large culm diameter of Takanari was responsible for the large SM. In contrast, Takanari had a small BS compared with that of Koshihikari. The FR was larger in Takanari than that in Koshihikari. The large FR in Takanari resulted from a large SMI. YM in Takanari was significantly higher than that in Koshihikari in 2015 but not in the following year.

**Table 1:** Breaking and bending type lodging resistance of Koshikari and Takanari in 2015 and 2016

Variety	Breaking type lodging resistance			Bending type lodging resistance		
	Bending moment at breaking gf·cm	Section modulus mm^3^	Bending Stress gf·mm^−2^	Flexural rigidity gf·mm^2^	Secondary moment of inertia mm^4^	Young’s modulus gf·mm^−2^
2015						
Takanari	1607	19.2	839.5	996.2	61.0	16.3
Koshihikari	1151	7.9	1462.3	226.4	18.2	12.4
	**	***	**	***	***	**
2016						
Takanari	1498	16.9	888.3	653.3	53.6	12.2
Koshihikari	953	6.8	1419.7	137.5	14.4	9.2
	**	***	**	**	***	ns

Note: ns, ** and *** indicated non-significant difference, significant at the level, 1 and 0.1% respectively

### Detected QTLs related to breaking- and bending-type lodging resistance on CSSLs in Takanari genetic background (T-CSSLs)

**Fig. 1:**
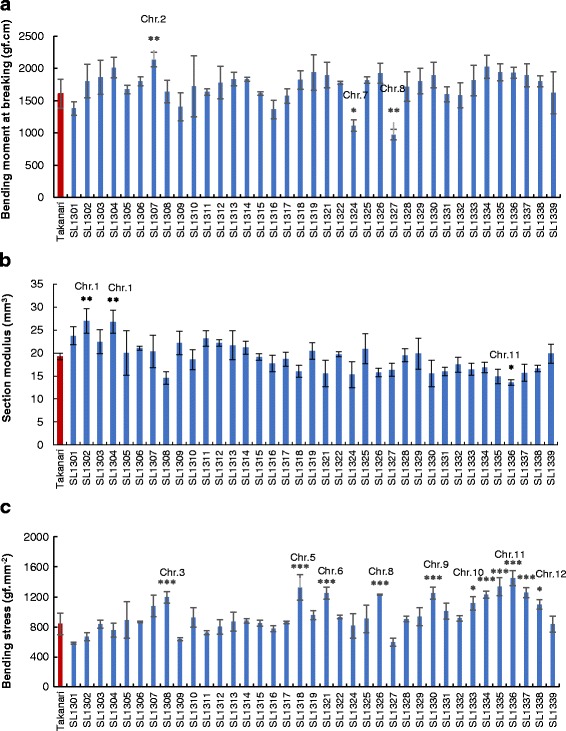
Physical parameters associated with breaking-type lodging resistance in T-CSSLs in 2015. **a** bending moment at breaking, **b** section modulus, and **c** bending stress of the fourth internode. Data are expressed as the mean ± SD. *, **, and *** represent significant differences at 5, 1, and 0.1% level, respectively

A total of 37 T-CSSLs of Koshihikari segments in the Takanari genetic background were investigated to identify QTLs related to bending- and breaking-type lodging resistance. Component traits of breaking-type and bending-type lodging resistance were compared with Takanari as the reference variety. A QTL was defined in CSSLs when a significant difference in related traits was observed between Takanari and the CSSLs. To detect QTLs for the parameters associated with breaking-type lodging resistance, a total of 37 T-CSSLs were compared with Takanari. Three T-CSSLs showed significant differences in the M compared with that in Takanari. One line (SL 1307) had a higher M value, and two lines (SL 1324 and SL 1327) showed lower M values than those of Takanari (Fig. 1a). The SM of Takanari was 19.2 mm^3^ and ranged from 27.0 mm^3^ to 13.5 mm^3^ among CSSLs (Fig. 1b). Significantly higher SM values were detected in SL 1302 and 1304 than that in Takanari, corresponding to substitutions of Koshihikari segments in the Takanari background on chromosome 1. By contrast, SL 1336 had a lower SM value than that of Takanari. The BS in Takanari was 839.5 gf·mm^− 2^; in CSSLs, BS values ranged from 583 gf·mm^− 2^ to 1451 gf·mm^− 2^. The BS values in SL 1308, 1318, 1321, 1326, 1330, 1333, 1334, 1335, 1336, 1337, and 1338 were significantly larger than that in Takanari (Fig. 1c).

**Fig. 2:**
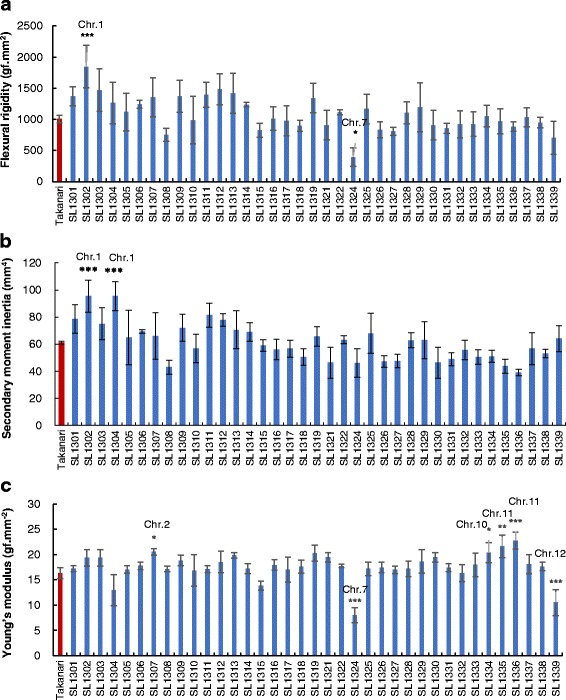
Physical parameters associated with bending-type lodging resistance in T-CSSLs. **a** flexural rigidity, **b** secondary moment inertia, and **c** Young’s modulus of the fourth internode. Data are expressed as the mean ± SD. *, **, and *** represent significant differences at 5, 1, and 0.1% level, respectively

To detect QTLs associated with bending-type lodging resistance, the basal culm of the fourth internode was compared between T-CSSLs and Takanari. The FR in Takanari was 996.2 gf ·mm^2^; in CSSLs, FR values ranged from 393.6 gf ·mm^2^ to 1847.3 gf ·mm^2^. The FR in SL 1302 was significantly higher than that in Takanari, whereas that in SL 1324 was smaller (Fig. 2a). The SMI values in two CSSLs (SL 130*2* and 1304) were significantly larger than that in Takanari (Fig. 2b). The YM in Takanari was 16.3 gf·mm^− 2^; in CSSLs, YM values ranged from 8.0 gf·mm^− 2^ to 22.7 gf·mm^− 2^. The YM was significantly smaller in SL 1324 and 1339 than that in Takanari, whereas the YM in SL 1307, 1334, 1335 and 1336 was larger (Fig. 2c).

### Cell wall materials in parents and CSSL lines

Cell wall materials contribute to culm stiffness, and therefore, identifying the component traits for cell wall materials to detect QTLs that affect the levels of these cell wall materials is important. In both years, the densities of cellulose and lignin in Takanari were higher than those in Koshihikari. No difference was detected in the hemicellulose densities of Takanari and Koshihikari in 2015, but Takanari showed a higher hemicellulose density than that of Koshihikari in 2016 (Table 2).

**Table 2:** Cell wall material density of Koshikari and Takanari in 2015 and 2016

Variety	Holocellulose density μg·mm^−3^	Lignin density μg·mm^−3^	Cellulose density μg·mm^− 3^	Hemicellulose density μg·mm^− 3^
2015				
Takanari	147.6	32.4	92.3	55.2
Koshihikari	107.7	26.1	66.2	41.4
	*	**	*	ns
2016				
Takanari	165.2	24.8	104.3	60.9
Koshihikari	123.3	9.8	85.8	37.6
	*	**	*	*

Note: ns, * and ** indicated, non-significant difference, significant at the level 5 and 1%

**Fig. 3:**
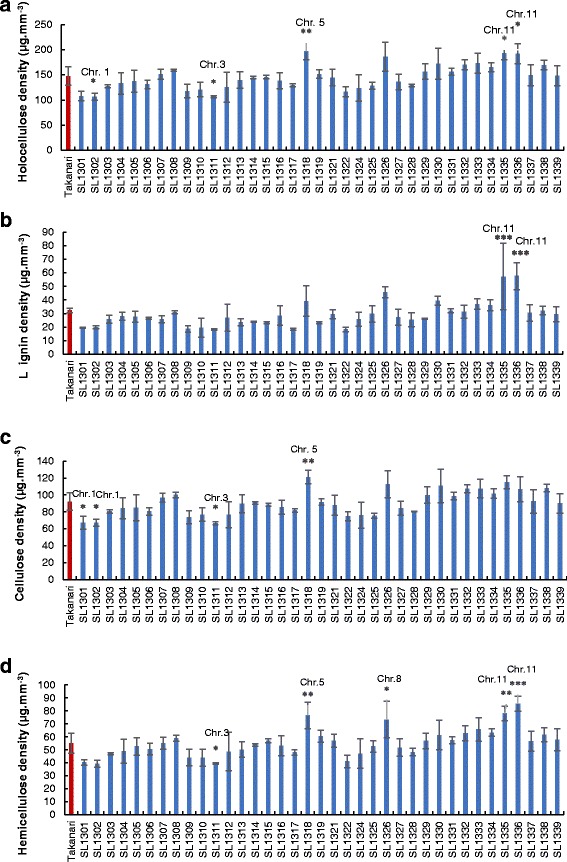
Cell wall material density of the fourth internode in 2015. **a** holocellulose, **b** lignin, **c** cellulose, and **d** hemicellulose. Data are expressed as the mean ± SD. *, **, and *** represent significant differences at 5%, 1, and 0.1% level, respectively

The densities of cell wall materials were compared between Takanari and 37 CSSLs (Fig. 3). The holocellulose density in Takanari was 147.6 μg·mm^− 3^; in T-CSSLs, holocellulose densities ranged from 106.3 μg·mm^− 3^ to 197.6 μg·mm^− 3^ (Fig. 3a). The cellulose density in Takanari was 92.4 μg·mm^− 3^, a value that was significantly higher than those in SL 1301, 1302, and 1311. One CSSL line, SL 1318, exhibited a significantly elevated cellulose density compared with that of Takanari (Fig. 3c). In five CSSLs, significant differences were detected in hemicellulose densities. Notably, SL 1311 exhibited a low hemicellulose density. In contrast, SL 1318, 1326, 1335 and 1336 had higher hemicellulose densities than that of Takanari (Fig. 3d).

### Substitution mapping of QTLs for component traits of lodging resistance

**Fig. 4:**
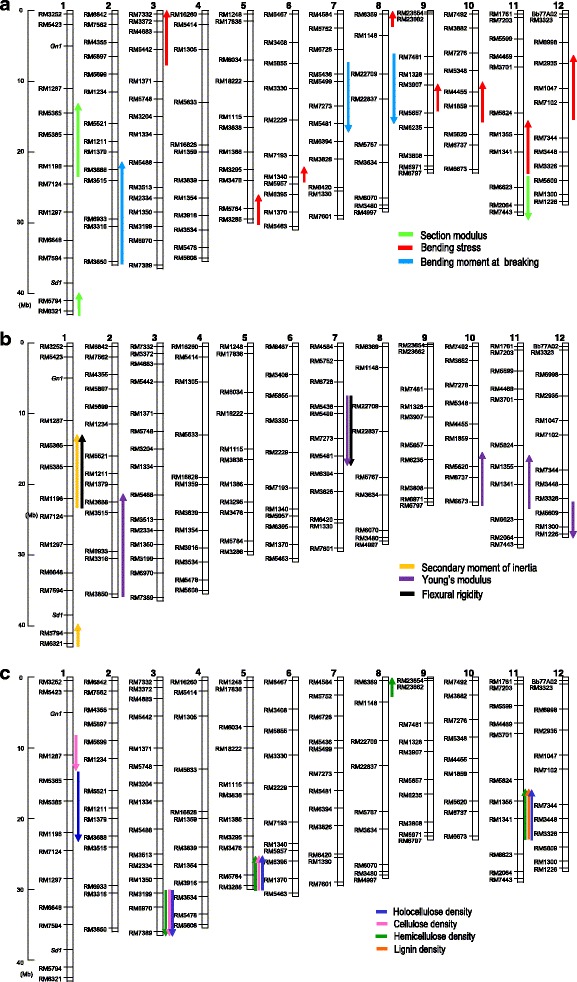
Substitution mapping of QTLs by comparing overlapped segments among T-CSSLs. **a** breaking-type lodging resistance, **b** bending-type lodging resistance, and **c** cell wall materials densities. Upward arrows indicate positive effects, and downward arrows indicate negative effects

To identify candidate regions for QTLs of cell wall materials associated with breaking- and bending-type lodging resistance, substitution mapping of the QTLs was conducted (Fig. 4). A total of 23 QTLs for breaking- and bending-type lodging resistance component traits were mapped. One QTL for M evaluated on chromosome 2 showed positive effects, whereas two QTLs on chromosome 7 and 8 showed a negative effect of the Koshihikari segment in the Takanari genetic background. Eight QTLs for BS were evaluated; all showed positive effects with the Koshihikari allele, and these QTLs were assigned to chromosomes 3, 5, 6, 8, 9, 10, 11, and 12 (Fig. 4a).

Two QTLs for FR were assigned to chromosomes 1 and 7. Two QTLs for SMI that were assigned to chromosome 1 showed a positive effect of the Koshihikari allele. Five QTLs were detected for YM; three and two of these QTLs contributed to increases and decreases, respectively, in YM as a result of the Koshihikari allele in the Takanari genetic background (Fig. 4b).

### Substitution mapping of QTLs for the traits responsible for culm strength associated with cell wall materials

Based on substitution mapping using T-CSSLs, a total of 12 QTLs were detected for regions associated with holocellulose, lignin, cellulose, and hemicellulose densities. The presence of a Koshihikari segment in the Takanari genetic background contributed to decreased cellulose density for QTLs on chromosomes 1 and 3. In contrast, the cellulose density increased by the placement of a Koshihikari segment on chromosome 5. Four QTLs were found for hemicellulose density, with three and one of these QTLs contributing to increases and decreases, respectively, in hemicellulose density (Fig. 4c).

QTLs for holocellulose, cellulose, and hemicellulose were estimated for the same regions as the one for BS on chromosome 5 and exhibited positive effects. These results suggested that the QTLs on chromosome 5 contributed to the increase in BS. On chromosome 11, QTLs for holocellulose, hemicellulose, and lignin were detected at the same region as those for BS and YM.

### QTLs for BS and cell wall materials associated with culm stiffness estimated using reciprocal CSSLs on chromosome 5

In this study, we detected QTLs for cell wall materials in the same region with BS on chromosomes 5 and 11 and in the same region with YM on chromosome 11. However, the performance of the CSSLs on chromosome 11 showed late maturity and abnormal growth compared with other lines (Additional file 4: Table S2 and Additional file 5: Table S3). Takai et al. (2014) reported that SL 1335 and 1336 showed hybrid weakness such as delayed heading, dwarf plant stature, fewer spikelets, lower ripening percentage and lower yield. Plant abnormality stature might affect the accumulation of cell wall materials, indirectly. Therefore, we focused on the QTL on chromosome 5.

**Fig. 5:**
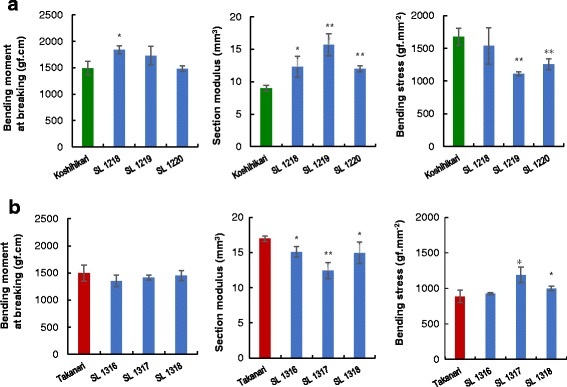
Physical parameters associated with breaking-type lodging resistance in **a** K-CSSLs and **b** T-CSSLs of chromosome 5 in 2016. Data are expressed as the mean ± SD. *, and ** represent significant differences at 5 and 1% level, respectively

To confirm the candidate regions of QTLs for the traits associated with BS and cell wall materials, reciprocal CSSLs of chromosome 5 were compared with the parent genetic background. Significant decreases in BS values were observed in K-CSSLs upon the substitution of Takanari segment into the Koshihikari genetic background. Two K-CSSLs, SL 1219 and 1220, exhibited smaller BS values than that of Koshihikari (Fig. 5a). By contrast, SL 1317 and SL 1318, lines with a reciprocal substitution at the same region, had a significantly higher BS than that of Takanari. Thus, a reciprocal effect between K-CSSLs and T-CSSLs on chromosome 5 was detected for BS (Fig. 5a and b).

**Fig. 6:**
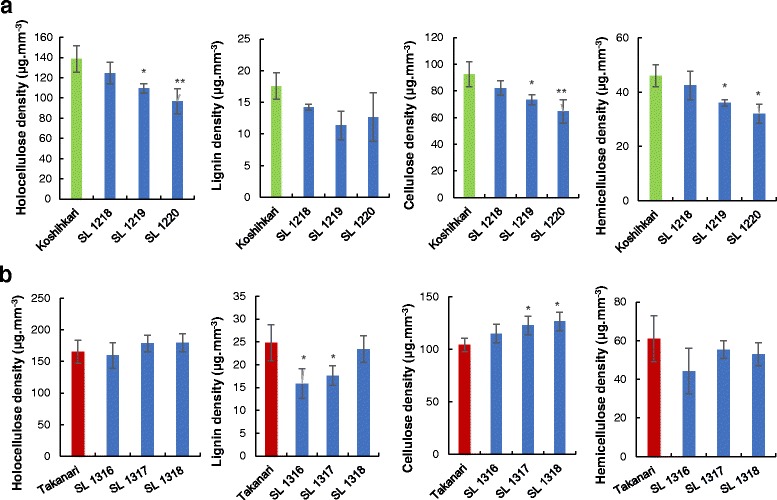
Cell wall material densities in **a** K-CSSLs and **b** T-CSSLs of chromosome 5 in 2016. Data are expressed as the mean ± SD. *, and ** represent significant differences at 5 and 1% level, respectively

Further investigations using reciprocal CSSLs were performed to confirm the results of QTLs for cell wall materials on chromosome 5. Two K-CSSLs, SL 1220 and 1219, showed low densities of holocellulose, cellulose, and hemicellulose when compared with Koshihikari (Fig. 6a). The cellulose density in SL 1317 and SL 1318 were higher than that in Takanari (Fig. 6b). The detected QTL at the long arm region on chromosome 5 was consistent with the result of the previous year in the Takanari genetic background (Fig. 3c).

**Fig. 7:**
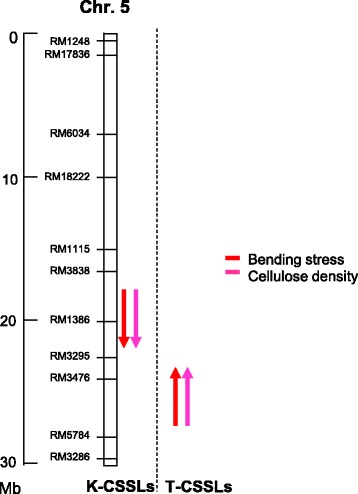
Substitution mapping of QTLs by comparing overlapped segments among lines of K-CSSLs and T-CSSLs for BS and cellulose density on chromosome 5 in 2016

QTLs for holocellulose, cellulose, and hemicellulose were assigned to the same regions on chromosome 5 as those detected for BS-associated QTLs on chromosome 5 in 2015 (Fig. 4a and c). In 2016, at the long arm region on chromosome 5, a reciprocal effect of T-CSSLs and K-CSSLs was observed for both BS and cellulose densities (Figs. 5a, b and 6a, b). This showed that the QTLs for BS and cellulose density were detected in the same region (Fig. 7) for each CSSLs background. These results suggested that the QTLs for cellulose density on chromosome 5 contributed to the increase BS in the Takanari genetic background.

## Discussion

### QTLs for the traits associated with breaking- and bending-type lodging resistance

CSSLs are ideal genetic populations in which to identify the traits that are controlled by many factors (Nadeau et al. 2000; Kubo et al. 2002; Ando et al. 2008). With CSSLs, phenotypic effects between alleles on substituted chromosome segments can be compared (Ebitani et al. 2005). Indeed, CSSLs have previously been used in rice to identify genes that control important traits related to lodging resistance (Kashiwagi 2014; Kashiwagi et al. 2016; Ookawa et al. 2016).

The parents of the CSSLs used here, Koshihikari and Takanari, have different lodging resistance characteristics. Koshihikari has a small culm diameter and a high BS due to the accumulation of lignin and cellulose in culms (Ookawa and Ishihara 1993), whereas the semi-dwarf *indica* variety, Takanari, has a large culm diameter (Xu et al. 1997; Ishikawa et al. 1999) and a small BS. Culm diameter is one of the traits that contribute to culm strength (Kashiwagi et al. 2008; Zuber et al. 1999; Ma et al. 2002; Sarker et al. 2007). Because SM is determined by culm diameter, Takanari, which has a large culm diameter, exhibits a large SM. The increases in culm diameter and culm wall thickness lead to high SM (Hirano et al. 2014).

In the present study, QTLs for SM were detected on chromosomes 1 and 11. In a previous study (Ookawa et al. 2016), a QTL for SM was detected on chromosome 1 and was located at the long arm in a region known to contain *SD1*. Thus, the QTL analysis in that study showed that the genomic region containing the *SD1/sd1* gene on chromosome 1 contributes to the regulation of SM. NIL-SD1 has a significantly larger SM than that of NIL *sd1* (Ookawa et al. 2016). The *sd1* allele provides significant increase in lodging resistance by decreasing the moment of the entire plant through the repression of internode elongation (Murai et al. 2004).

The QTL for SM was detected at the long arm region on chromosome 1 in this study (Fig. 1b), consistent with the results of previous study using the same reciprocal CSSLs (Ookawa et al. 2016). The Koshihikari allele of *SD1* contributes to the thick culm, although Koshihikari exhibits a fine culm.

Reciprocal CSSLs confer the advantages of enabling evaluation of differences in the allelic effect of QTLs in both genetic backgrounds. When detected QTLs in both genetic backgrounds show reciprocal effects, these loci should have no genetic interaction or epistasis with other background factors (Kubo et al. 2002). The SM-associated QTL on chromosome 5 showed a reciprocal effect at the same region in reciprocal CSSLs. The SM in CSSLs was increased by replacement of the Takanari allele in the Koshihikari genetic background (Fig. 5a), and in the reciprocal lines. The SM decreased with the Koshihikari allele in the Takanari genetic background (Fig. 5b). These results indicated that the same allele on chromosome 5 is responsible for regulating SM. A previous study using the same CSSLs showed that SM is increased by the Takanari allele in this region and decreased by the Koshihikari allele in the reciprocal line (Ookawa et al. 2016). Takai et al. (2014) observed a similar reciprocal effect for yield component traits and photosynthetic rate.

QTLs for BS were identified on chromosomes 3, 5, 6, 8, 9, 10, 11, and 12. These QTLs contributed to an increase in BS when the Koshihikari chromosomal segment was substituted into the Takanari genetic background. Using the same reciprocal CSSL, Ookawa et al. (2016) also detected QTLs for BS on chromosomes 6, 8, and 11. Thus, the results of both studies showed that these QTLs contributed to an increase in BS. Some QTLs for BS were not detected in the previous study (Ookawa et al. 2016). This discrepancy might be caused by the complexity of the BS trait. BS is a complex trait controlled by many responsible traits such as morphological traits and cell wall materials (Matsuda et al. 1983) and by environmental factors, which might explain why some QTLs were detected at the different regions, reciprocally.

QTLs for BS and cellulose density were estimated at the same regions in K-CSSLs and T-CSSLs, respectively. However, the estimated region in K-CSSLs did not overlap with that in T-CSSLs (Fig. 7). Estimating the different regions of QTLs in the reciprocal genetic backgrounds might be explained by the existence of multiple genes with the same function in the segment as a QTL cluster or epistasis with genetic background (Matsubara et al. 2016). Further study is required to narrow down and identify the candidate genes associated with BS and cellulose density.

In the present study, we also detected QTLs related to bending-type lodging resistance. Bending-type lodging resistance is defined by FR values indicating culm stiffness. FR is composed of YM and SMI. YM depends on the composition of plant tissue, and SMI depends on the configuration of existing material, i.e., culm outer and inner diameters (Silk et al. 1982). Large differences in YM were found between the Koshihikari and Takanari. YM is an indicator of the rigidity of rice culm (Ishimaru et al. 2008). YM was increased by the substitution of Koshihikari chromosomal segments on chromosomes 2, 10, and 11. Other QTLs that were identified on chromosomes 7 and 12 indicated that Koshihikari alleles contributed to the decrease of YM in the Takanari genetic background. This result suggested that the detected regions included the genes responsible for the elevation of YM in Takanari.

QTLs for BS, YM and cell wall materials were detected at the same regions on chromosomes 5 and 11 (Fig. 4a, b and c). The CSSLs on chromosome 11 had the characters of later maturity and short stature compared with those in other lines, which indicated that this phenotype affected the accumulation of cell wall materials indirectly. These phenotypes might be caused by the *hdb3* allele from Koshihikari located in the long arm region on chromosome 11. This gene has a function of hybrid breakdown (Yamamoto et al. 2007). Hybrid breakdown (sterility or weakness in later generations) is commonly observed in crosses between *indica* and *japonica* (Oka 1988; Matsubara et al. 2007; Yamamoto et al. 2007). In rice, hybrid breakdown causes reduced tiller numbers, retarded growth with short culm and panicle, chlorosis of leaves, poor seed set and retarded root growth (Jiang et al. 2008; Sunohara et al. 2009). This result explains why we only focused on the QTLs for cell wall materials on chromosome 5.

### QTLs for cell wall materials associated with culm stiffness

The detection of multiple QTLs for different traits may facilitate an improved strategy for developing lines with increased lodging resistance. Ookawa et al. (2010a) showed that the Leaf Star has superior lodging resistance characteristics because of culm thickness and culm stiffness traits. The combination of multiple QTLs with different functions improved performance. Notably, the pyramiding of lines carrying *SCM2* and *SCM3* yielded much stronger culm performance than either QTL alone (Yano et al. 2015). The combination of *BSUC11* and a QTL for non-structural carbohydrate (NSC) increased lodging resistance, presumably by improving the chemical component(s) in culm (Kashiwagi et al. 2016). In rice, culm morphological features contribute to culm strength, and some biochemical characteristics (such as the levels of cellulose, hemicellulose, holocellulose, and lignin) are also important for lodging resistance (Kashiwagi and Ishimaru 2004; Li et al. 2008). Kokubo et al. (1989, 1991) reported that cellulose content correlated with BS in barley and that the *brittle culm* (*bc*) mutant showed low cellulose content due to decreased cellulose biosynthesis in the cell wall. The accumulation of cellulose, hemicellulose, and lignin improves cell wall thickness and flexibility (Kong et al. 2013). A study by Kashiwagi et al. (2016) showed that holocellulose is the primary culm component that is responsible not only for culm strength but also for prevention of culm strength deterioration. Ookawa et al. (1993) reported that the Koshihikari line and most *japonica* varieties have high BS, a phenotype caused by elevated levels of cellulose and lignin in the culm.

We detected QTLs on chromosome 5 that related to BS and the density of cellulose; both revealed positive effects for the Koshihikari allele. The QTLs for cellulose density and BS were confirmed in reciprocal CSSLs. This result indicated that the Koshihikari allele on chromosome 5 had a positive effect on cellulose density, although the Koshihikari accumulated cellulose to a level lower than that in Takanari. Ookawa et al. (2016) observed a similar mechanism and showed that the Koshihikari, a long-culm *japonica* variety, harbours the superior allele of the chromosome-1 *SD1* gene. Although the Koshihikari line has a fine culm, this line harbours a thick-culm *SD1* allele. These results suggested that the *japonica*-variety Koshihikari has a hidden superior allele on chromosome 5 that contributes to the improved culm stiffness and lodging resistance of *indica* varieties such as Takanari.

In the present study, we detected QTLs for strong culm traits related to breaking- and bending-type lodging resistance. We found a new QTL for cellulose density that mapped to the long arm of chromosome 5. This result suggested that the BS of the Takanari *indica* rice variety could be improved by introducing the allele for increased cellulose density from the *japonica*-variety Koshihikari. Further study is required to narrow the region and identify the responsible genes; such results are expected to increase our understanding of the genetic factors controlling the development of rice varieties with improved bending- and breaking-type lodging resistance.

## Conclusions

BS was increased by substitution of the Koshihikari chromosomal segments in the Takanari genetic background. Koshihikari allele on chromosome 5 contributed to the increase of BS. QTLs for cellulose density were also estimated on chromosome 5, which were replaced by substitution of Koshihikari segments. The QTLs for hemicellulose, cellulose, and holocellulose densities identified on chromosome 5 overlapped with those for BS, indicating the positive effect of the Koshihikari segment on increasing BS. These results suggested that the QTLs for the densities of cell wall materials in *japonica* varieties contributed to increased BS and could be utilized for improving lodging resistance in *indica* rice varieties.

## Additional files

Additional file 1: **Figure S1.** Graphical genotypes of the reciprocal CSSLs. (a) 41 K-CSSLs, (b) 39 T-CSSLs. Orange regions indicate homozygosity for Koshihikari; blue regions indicate homozygosity for Takanari. Gray region indicates heterozygosity. Genotypes of the 141 SSR markers in both CSSLs are shown in the upper parts of graphs. A: Koshihikari genotype, B: Takanari genotype. (PPTX 1392 kb)

Additional file 2: **Figure S2.** Graphical genotypes of the reciprocal CSSLs of chromosome 5 (a) K-CSSLs, (b) T-CSSLs. Orange regions indicate homozygosity for Koshihikari; blue regions indicate homozygosity for Takanari. (DOCX 18 kb)

Additional file 3: **Table S1.** Analysis of variance of the influence of lines on breaking type lodging and cell wall composition densities of chromosome 5 in 2016. (DOCX 13 kb)

Additional file 4: **Table S2.** Heading date (date after sowing) of parent lines and T-CSSLs in 2015. (PPTX 291 kb)

Additional file 5: **Table S3.** Heading date (date after sowing) of parent lines and reciprocal CSSLs of chromosome 5 in 2016. (DOCX 19 kb)

## Abbreviations

BS: Bending stress
CSSLs: Chromosome segment substitution lines
FR: Flexural rigidity
GA: Gibberellin
K-CSSLs: CSSLs in the Koshihikari genetic background
M: Bending moment at breaking
NIL: Near isogenic lines
NSC: Non-structural carbohydrate
QTL: Quantitative trait loci
SCM: Strong culm
SD1/sd1: Semi-dwarf1
SM: Section modulus
SMI: Secondary moment of inertia
T-CSSLs: CSSLs in the Takanari genetic background
YM: Young’s modulus
