# Supplementary material for: Finding the superior allele of japonica-type for increasing stem lodging resistance in indica rice varieties using chromosome segment substitution lines
Source: Rice (N Y). 2018 Apr 18;11:25. doi: 10.1186/s12284-018-0216-3 (PMC5906422; doi:10.1186/s12284-018-0216-3)
Supplement: Supplementary file 1 — Figure S1. Graphical genotypes of the reciprocal CSSLs. (a) 41 K-CSSLs, (b) 39 T-CSSLs. Orange regions indicate homozygosity for Koshihikari; blue regions indicate homozygosity for Takanari. Gray region indicates heterozygosity. Genotypes of the 141 SSR markers in both CSSLs are shown in the upper parts of graphs. A: Koshihikari genotype, B: Takanari genotype. (PPTX 1392 kb) [file 12284_2018_216_MOESM1_ESM.pptx]

## Slide 1
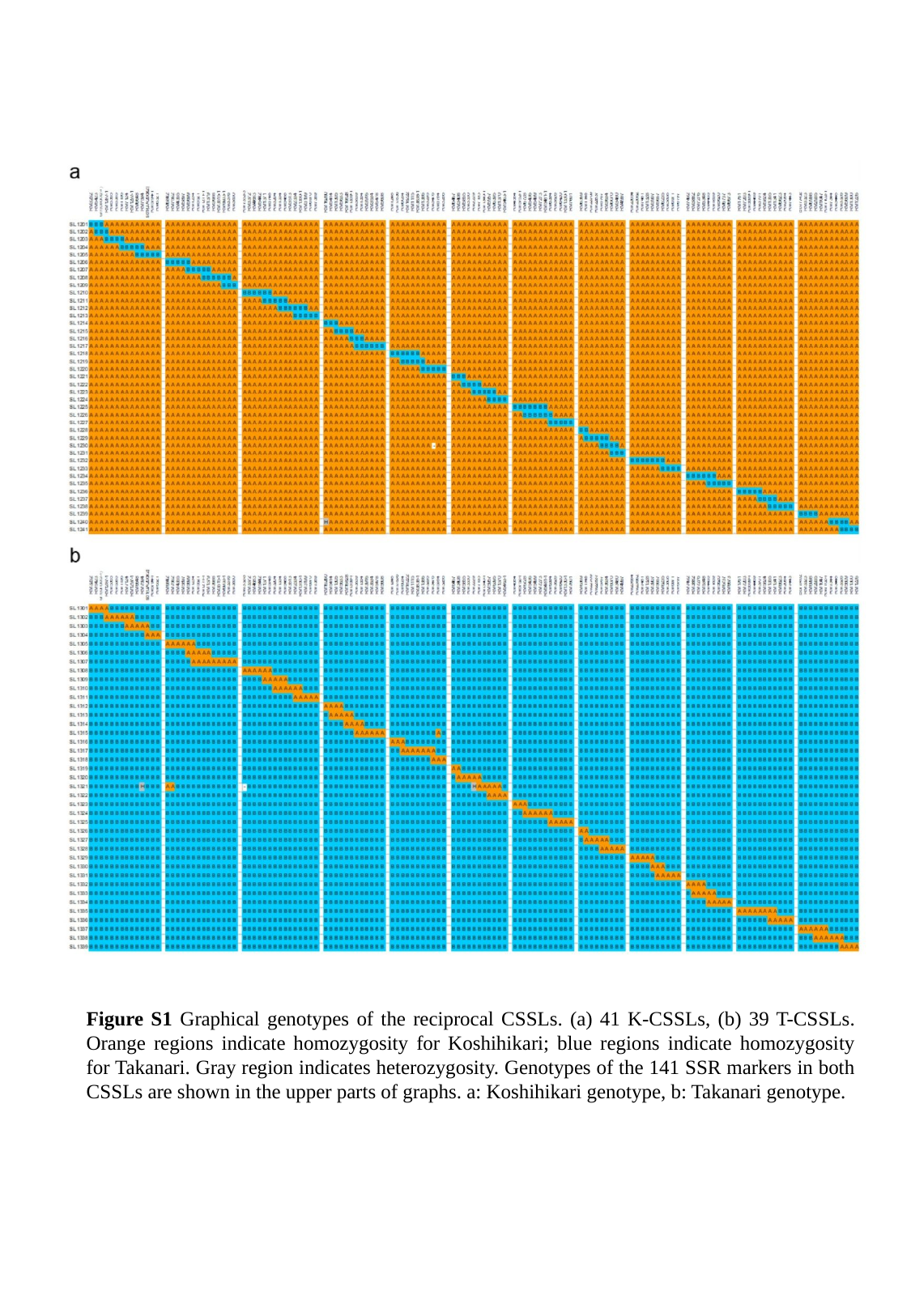

Figure S1 Graphical genotypes of the reciprocal CSSLs. (a) 41 K-CSSLs, (b) 39 T-CSSLs. Orange regions indicate homozygosity for Koshihikari; blue regions indicate homozygosity for Takanari. Gray region indicates heterozygosity. Genotypes of the 141 SSR markers in both CSSLs are shown in the upper parts of graphs. a: Koshihikari genotype, b: Takanari genotype.
