# Supplementary material for: Finding the superior allele of japonica-type for increasing stem lodging resistance in indica rice varieties using chromosome segment substitution lines
Source: Rice (N Y). 2018 Apr 18;11:25. doi: 10.1186/s12284-018-0216-3 (PMC5906422; doi:10.1186/s12284-018-0216-3)
Supplement: Supplementary file 2 — Figure S2. Graphical genotypes of the reciprocal CSSLs of chromosome 5 (a) K-CSSLs, (b) T-CSSLs. Orange regions indicate homozygosity for Koshihikari; blue regions indicate homozygosity for Takanari. (DOCX 18 kb) [file 12284_2018_216_MOESM5_ESM.docx]

**Table S3**. Heading date (date after sowing) of parent lines and reciprocal CSSLs of

chromosome 5 in 2016.

| Lines | Heading  (days after sowing) | Lines | Heading  (days after sowing) |
| --- | --- | --- | --- |
| Koshihikari | 97 | Takanari | 103 |
| SL 1218 | 98 | SL 1316 | 103 |
| SL 1219 | 94 | SL 1317 | 107 |
| SL 1220 | 96 | SL 1318 | 100 |
