# Supplementary material for: Finding the superior allele of japonica-type for increasing stem lodging resistance in indica rice varieties using chromosome segment substitution lines
Source: Rice (N Y). 2018 Apr 18;11:25. doi: 10.1186/s12284-018-0216-3 (PMC5906422; doi:10.1186/s12284-018-0216-3)
Supplement: Supplementary file 3 — Table S1. Analysis of variance of the influence of lines on breaking type lodging and cell wall composition densities of chromosome 5 in 2016. (DOCX 13 kb) [file 12284_2018_216_MOESM3_ESM.docx]

**Table S1.** Analysis of variance of the influence of lines on breaking type lodging and cell wall composition densities of chromosome 5 in 2016.

| source of variation | M (gf.cm) | SM (mm^3^) | BS (gf.mm^-2^) | Holocellulose density (µ.mm^-3^) | Lignin density (µ.mm^-3^) | Cellulose density (µ.mm^-3^) | Hemicellulose density (µ.mm^-3^) |
| --- | --- | --- | --- | --- | --- | --- | --- |
| Koshihikari and K-CSSLs | * | ** | ** | ** | ns | ** | * |
| Takanari and T-CSSLs | ns | ** | ** | ns | * | * | ns |
