# Supplementary material for: Finding the superior allele of japonica-type for increasing stem lodging resistance in indica rice varieties using chromosome segment substitution lines
Source: Rice (N Y). 2018 Apr 18;11:25. doi: 10.1186/s12284-018-0216-3 (PMC5906422; doi:10.1186/s12284-018-0216-3)
Supplement: Supplementary file 4 — Table S2. Heading date (date after sowing) of parent lines and T-CSSLs in 2015. (PPTX 291 kb) [file 12284_2018_216_MOESM2_ESM.pptx]

## Slide 1
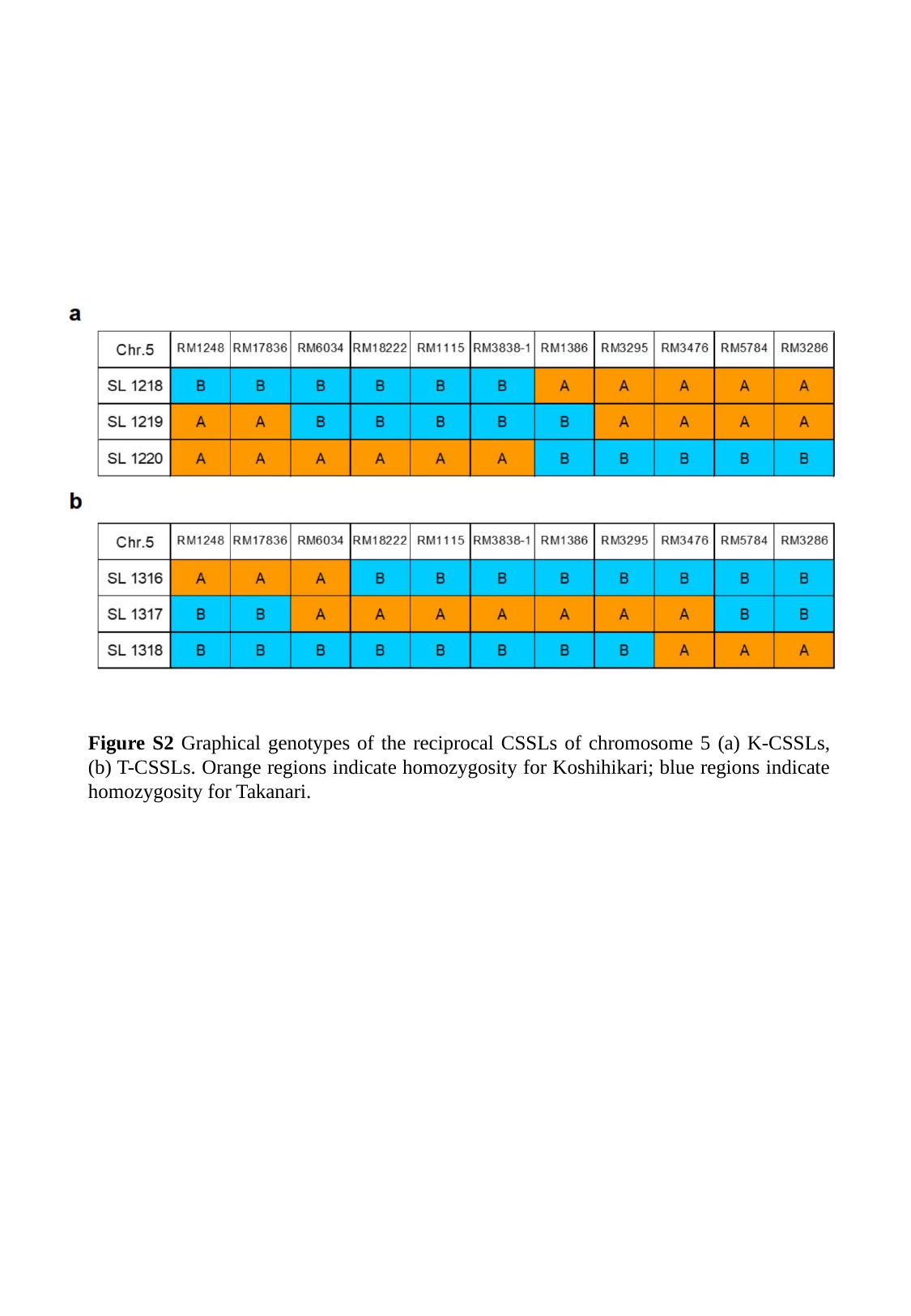

Figure S2 Graphical genotypes of the reciprocal CSSLs of chromosome 5 (a) K-CSSLs, (b) T-CSSLs. Orange regions indicate homozygosity for Koshihikari; blue regions indicate homozygosity for Takanari.
