# Supplementary material for: Finding the superior allele of japonica-type for increasing stem lodging resistance in indica rice varieties using chromosome segment substitution lines
Source: Rice (N Y). 2018 Apr 18;11:25. doi: 10.1186/s12284-018-0216-3 (PMC5906422; doi:10.1186/s12284-018-0216-3)
Supplement: Supplementary file 5 — Table S3. Heading date (date after sowing) of parent lines and reciprocal CSSLs of chromosome 5 in 2016. (DOCX 19 kb) [file 12284_2018_216_MOESM4_ESM.docx]

**Table S2**. Heading date (date after sowing) of parent lines and T-CSSLs in 2015.

| Lines | Heading  (days after sowing) | Lines | Heading  (days after sowing) |
| --- | --- | --- | --- |
| Koshihikari | 96 | SL 1319 | 107 |
| Takanari | 105 | SL 1321 | 98 |
| SL 1301 | 103 | SL 1322 | 109 |
| SL 1302 | 103 | SL 1324 | 91 |
| SL 1303 | 102 | SL 1325 | 92 |
| SL 1304 | 95 | SL 1326 | 95 |
| SL 1305 | 92 | SL 1327 | 105 |
| SL 1306 | 98 | SL 1328 | 111 |
| SL 1307 | 107 | SL 1329 | 105 |
| SL 1308 | 100 | SL 1330 | 98 |
| SL 1309 | 103 | SL 1331 | 100 |
| SL 1310 | 109 | SL 1332 | 107 |
| SL 1311 | 91 | SL 1333 | 95 |
| SL 1312 | 91 | SL 1334 | 97 |
| SL 1313 | 107 | **SL 1335** | **127** |
| SL 1314 | 107 | **SL 1336** | **131** |
| SL 1315 | 107 | SL 1337 | 95 |
| SL 1316 | 102 | SL 1338 | 95 |
| SL 1317 | 109 | SL 1339 | 91 |
| SL 1318 | 92 |  |  |
